# Cyclic polyglycolide by means of metal acetylacetonates

**DOI:** 10.1039/d5ra07961f

**Published:** 2026-01-06

**Authors:** Steffen M. Weidner, Andreas Meyer, Jana Falkenhagen, Hans R. Kricheldorf

**Affiliations:** a Bundesanstalt für Materialforschung und -prüfung–BAM Richard Willstätter Strasse11 D-12489 Berlin Germany; b Universität Hamburg, Institut für Physikalische Chemie Grindelallee 117 Hamburg D-20146 Germany; c Universität Hamburg, Institut für Technische und Makromolekulare Chemie Bundesstrasse 45 D-20146 Hamburg Germany hrkricheldorf@aol.de

## Abstract

Glycolide was polymerized in bulk at 160 °C using various metal acetylacetonates as catalysts. Zirconium acetylacetonate was particularly efficient, enabling rapid polymerization even at 130 °C. The formation of cyclic poly(glycolic acid) (PGA), most likely *via* a ring-expansion polymerization (REP) mechanism, was proven by matrix-assisted laser desorption/ionization time-of-flight (MALDI-TOF) mass spectrometry. Depending on the polymerization conditions, the formation of even-numbered cycles was favored to varying degrees. Number-average molecular weights (*M*_n_) in the range of 2000–3500 g mol^−1^ were achieved with dispersities below 2.0. Wide-angle X-ray scattering (WAXS) powder patterns showed that the crystal lattice was the same as that of known linear PGAs, regardless of the *M*_n_ values. These patterns enabled a comparison of crystallinities with values derived from DSC measurements.

## Abstract 2

Polymerizations of glycolide with various metal acetylacetonates showed that Zr(acac)_4_ is the most reactive catalyst, yielding low-molecular-weight cyclic poly(glycolide). The combination of WAXS and DSC measurements confirmed a Δ*H*^0^_m_ of 206 J g^−1^.

## Graphical abstract

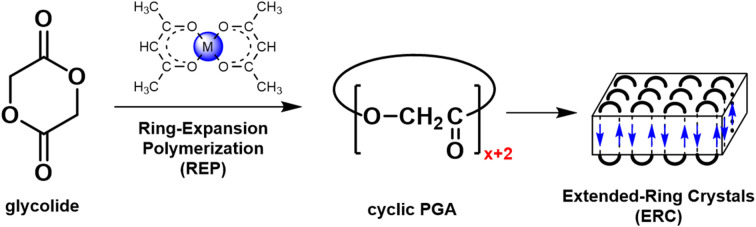

## Introduction

Interest in the synthesis and characterization of polyglycolide (PGA) increased among academics and industry members when, in 1952/53, members of American Cyanamide and DuPont filed patents describing the synthesis and properties of this biodegradable polyester, mentioning its potential usefulness in medical applications.^1–3^ These initial patents were followed by a relatively slow but steady stream of additional patents and academic research articles.^4–27^ In 1968, American Cyanamide commercialized PGA fibres under the trademark “Dexon' for use as a resorbable medical suture. A few years later, Ethicon Inc., a subsidiary of Johnson & Johnson, followed with the commercialization of a medical suture under the trademark “Vicryl”. This suture is made from a copolyester of glycolic and lactic acid, containing more than 90% glycolic acid. These two sutures are still widely used in the 21st century. Over the past thirty years, further applications have emerged, such as films for packaging purposes under the trademark “Kuradex.”

PGA has a unique combination of properties. The absence of substituents enables PGA to form a compact crystal lattice through the parallel arrangement of linear chains in a planar zigzag conformation. This results in a structure resembling the β-sheet structure of polyglycine. This dense chain packing results in a high-density crystalline PGA with a specific density that can reach values around 1.69 g cm^−3^ and a hardness that is relatively high compared to other aliphatic polyesters.6 Another corollary is a stable crystal lattice with a high melting temperature (*T*_m_ up to 232 °C) and a high melting enthalpy (theoretical Δ*H*^0^_m_ = 206 J g^−1^).^14,20^ The tight chain packing also provides excellent barrier properties against gases, particularly non-polar ones. These properties, combined with high mechanical strength, make PGA an interesting material for food packaging. However, applications and research papers dealing with PGA are scarce compared to those dealing with polylactide because PGA has a few negative properties. First, glycolide polymerization, the standard technical procedure, is highly exothermic, and controlling the heat flow is difficult when polymerizing several tons of material. Second, processing from the melt requires temperatures above 230 °C, which pose a high risk of thermal degradation and discoloration. The most significant shortcoming for the technical production and commercialization of PGA, however, is the high cost of the monomer. Nonetheless, copolyesters of glycolide and l-lactide (and sometimes a third comonomer) have widespread applications as drug delivery systems because the small quantities needed, and the high cost of drugs mean the cost of glycolide does not matter. Hundreds of papers and patents have been published in this field. All these commercial homopolymers and copolymers of glycolic acid have a linear structure resulting from alcohol-initiated polymerizations of GL. Over the past 50 years, interest in cyclic polymers has dramatically increased because their properties deviate from those of linear polymers in various ways.^28–35^ In the case of poly(l-lactide), another biodegradable polyester with a broader range of applications than PGA, more than 60 papers dealing with the synthesis and characterization of cyclic poly(l-lactides) (cPLA) have appeared in the last 20 years. Most of these papers are cited in ref. 36–38, but a comprehensive review is lacking. However, by the end of 2023, no publications concerning the synthesis and characterization of cyclic PGA (cPGA) had appeared. In subsequent years, the authors published two papers describing the synthesis of high-molecular-weight cPGAs using tin catalysts with number-average molecular weights (*M*_n_) ranging from 100 000 to 300 000.^39,40^ Recently, the authors demonstrated that extremely low-molecular-weight cPGAs (*M*_n_ < 1000 g mol^−1^) can be prepared *via* zwitterionic polymerization of GL with pyridine.^41^

**Scheme 1:**
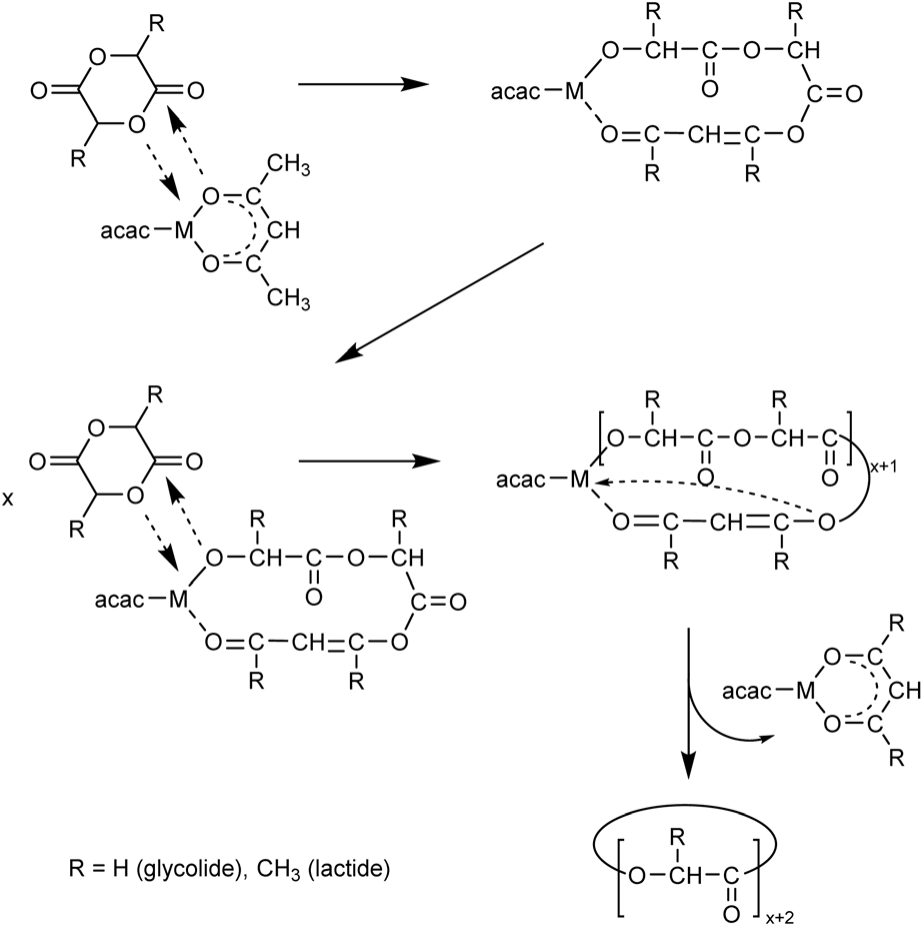
REP mechanism of GL and LA polymerizations catalyzed by a metal acac-complex.

However, none of these methods allows control of the molecular weight *via* the monomer/catalyst ratio. A broad variation of molecular weights is desirable for potential applications and academic studies. A synthetic approach that yields cPGAs with *M*_n_ values ranging from 1000 to 100 000 g mol^−1^ is still lacking, and this gap can only be closed by varying the catalyst and reaction conditions. This situation prompted the authors to examine GL polymerizations with metal acetylacetonate complexes for two reasons. First, these complexes are commercially available, enable polymerization in bulk at high temperatures, and are usually insensitive to air and stable during storage. Second, the authors recently discovered that Zr(acac)_4_ enables the synthesis of cyclic PLAs with *M*_n_ values in the range of 20 000–75 000 g mol^−1^ most likely *via* the ring-expansion polymerization mechanism outlined in Scheme 1. An additional advantage of this catalyst is its extremely low toxicity. Therefore, the purpose of this study was to examine the potential of metal acac complexes as catalysts for synthesizing cPGAs with *M*_n_'s ranging from 1000 to 100 000 g mol^−1^.

## Experimental

### Materials

Glycolide was purchased from PhyScience (Hirschberg, Germany) and recrystallized from hot anhydrous THF (Fisher Scientific, Schwerte, Germany) by gradual addition of toluene (Fisher Scientific) and partial removal of THF *in vacuo*. THF and toluene were used as received. The acetylacetonate complexes of manganese(ii), vanadyl oxide, dibutyltin and zirconium(iv) were purchased from Merck/Sigma-Aldrich and used as received with exception of Zr(acac)_4,_ which was recrystallized from toluene.

### Polymerizations

(a) with various metal acac-complexes (Table 1)

**Table 1:** ROPs of glycolide catalyzed by various metal acetylacetonates at 160 °C in bulk^a^

Exp. no.	Catalyst	Temp. [°C] =	Time [min]	*M*_n_ [g mol^−1^]	*M*_w_	Dispersity *Đ*	*T*_m_ [°C]	Δ*H*_m_ [J g^−1^]
1	Bu_2_Sn(acac)_2_	160	30	2250	2900	1.3	213.7	91.4
2	Mn(acac)_2_	160	30	2500	3350	1.3	217.6	104.0
3	Ni(acac)_2_	160	150	Insoluble	—	—	211.8	88.2
4	VO(acac)_2_	160	120	Insoluble	—	—	203.3	89.8
5	Cu(acac)_2_	160	120	low conversion	—	—	—	—

a Calculated with Δ*H*^0^_m_ = 206 J g^−1^.

Glycolide (40 mmol) and a metal acac complex (0.08 mmol) were weighed into a flame-dried 40 mL round-bottom flask under a blanket of argon. Then, a magnetic bar was added. The reaction vessel was immersed into an oil bath preheated to 160 °C. After cooling, the porous solid cake of PGA was broken up with a spatula removed from the reaction vessel and characterized in the virgin state.

(b) with Zr(acac)_4_ (GL/Cat = 500/1) (Table 2)

Glycolide (40 mmol) and Zr(acac)_4_ (0.08 mmol) were weighed into a flame-dried 50 mL round-bottom flask under a blanket of argon. Then, a magnetic bar was added. The reaction vessel was immersed in an oil bath preheated to either 130 °C or 160 °C, and the reaction was carried out as described above.

Experiments with other GL/Cat ratios were performed analogously.

### Measurements

The MALDI TOF mass spectra were measured with an Autoflex maX mass spectrometer (Bruker Daltonik GmbH, Bremen, Germany) equipped with a Smartbeam laser. The linear positive ion mode was used. The MALDI stainless steel targets were prepared from solutions of PGA and matrix in 1,1,1,3,3,3-hexafluoroisopropanol (HFIP) and doped with potassium trifluoroacetate (2 mg mL^−1^). Typically, 20 µL of the sample solution (5 mg mL^−1^), 2 µL of the potassium salt solution and 50 µL of a solution of *trans*-2-[3-(4-*tert*-butylphenyl)-2-methyl-2-propenylidene] malononitrile (DCTB, 20 mg mL^−1^ in HFIP) were mixed in an Eppendorf vial. 1 µL of the corresponding solution was spotted on the stainless-steel target. FlexControl (Bruker Daltonik GmbH) was used to record spectra by accumulating 4 × 2000 single laser shots were recorded at 4 different positions.

Electrospray ionization (ESI) TOF MS measurements were performed using a Q-TOF Ultima ESI-TOF mass spectrometer (Micromass), which was set to a capillary voltage of 4 kV, a source temperature of 120 °C and a desolvation temperature of 150 °C. The mass spectrometer was operated in the positive ion mode. The sample (0.1 mg mL ^−1^) was dissolved in HFIP. To improve ionization, 10 µL of a 0.1 mg L^−1^ potassium trifluoroacetate solution was added to 1 mL of the sample solution. Data were processed using MassLynx 4.1 (Waters). To generate a spectrum, scans were accumulated for 0.5 min (scan time 1 min; up to 100 000 spectra per second). Here, only the lower mas range up to *m*/*z* 1500 was considered.

The gel permeation chromatography (GPC) measurements were performed in a modular system kept at 30 °C consisting of an isocratic pump (Agilent, USA) running with a flow rate of 0.5 mL min^−1^ and a refractive index detector (RI-501-Shodex). HFIP was used as eluent. Samples were manually injected (100 µL, *ca.* 2–4 mg mL^−1^). For instrument control and data calculation WinGPC software (Polymer Standard Service-PSS now Agilent, Mainz, Germany) was used. The calibration was performed using a polymethylmethacrylate (PMMA) standard set (Polymer Standards Service–PSS, Mainz).

The DSC heating traces were recorded on a (with indium and zinc freshly calibrated) Mettler-Toledo DSC-1 equipped with Stare Software-11 using a heating rate of 10 K min^−1^. Only the first heating traces were evaluated. The crystallinities were calculated with a Δ*H*_m_ max of −206 J g^−1^.

The small-angle X-ray scattering (SAXS) and WAXS measurements were conducted at the Institute of Physical Chemistry, University of Hamburg. The self-designed SAXS/WAXS apparatus, equipped with an Incoatec™ X-ray source IµS with Quazar Montel optics and scatterless pinholes has a focal spot size diameter at sample of 700 µm at a wavelength of 0.1542 nm. An evacuated flight tube with distance between sample and detector of 1.4 m was installed for the SAXS measurements. A Hybrid-Pixel-Detector Dectris™ Eiger2 1 M was employed for detection. The regular measurement time per sample accounted for 10 min. DPDAK, a customizable software for reduction and analysis of X-ray scattering data sets was used for gathering 1D scattering curves.^42^ For the evaluation of the crystallinity of the samples the data were imported in Origin™ and analyzed with the peak analysis module. The long periods of the lamellar domains were determined by the *q* values of the reflection maxima of the SAXS curves.

## Results and discussion

### Polymerizations with various metal acetylacetonates

**Fig. 1:**
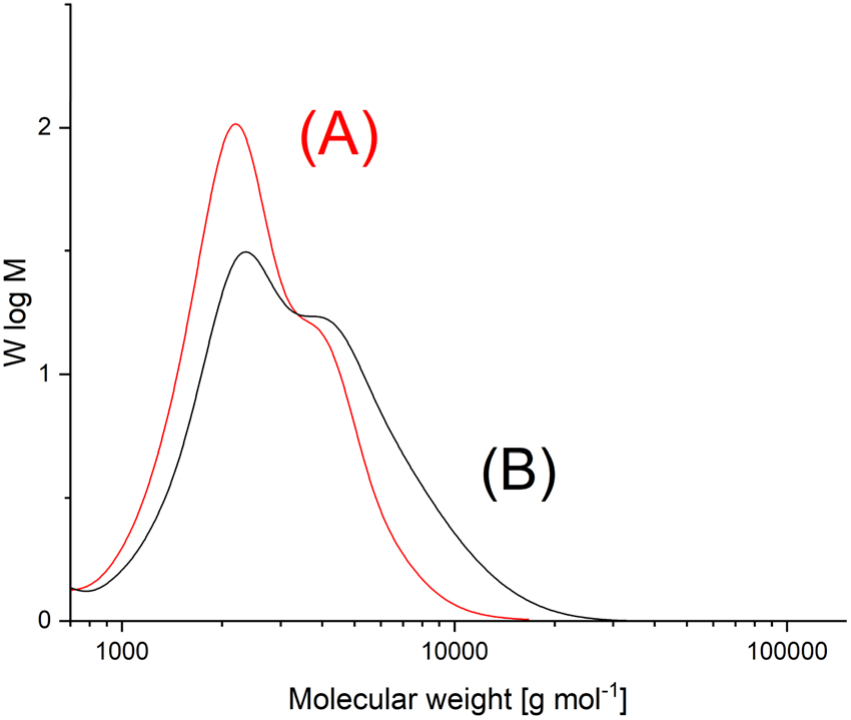
GPC mass distribution curves of cPGA prepared (A) with Bu_2_Sn(acac)_2_ in bulk at 160 °C (No. 1, Table 1), (B) With Zr(acac)_4_ (GL/cat = 500/1) in bulk at 160 °C/15 min (No. 2, Table 2).

**Fig. 2:**
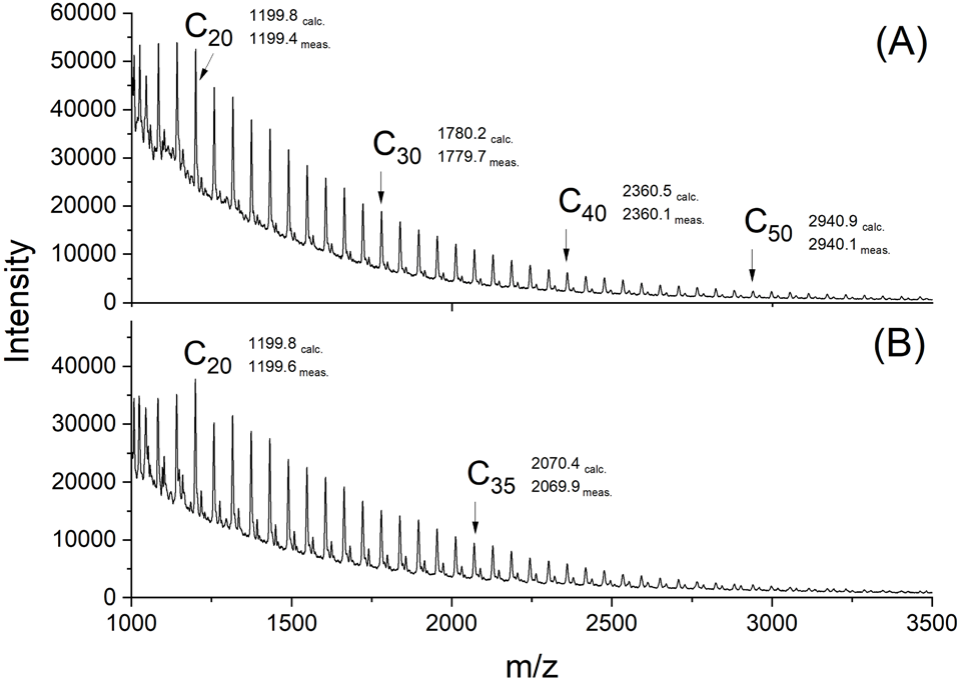
MALDI TOF mass spectra of cPGAs (A) prepared with Bu_2_Sn(acac)_2_ in bulk at 160 °C (No.1, Table 1); (B) prepared with Zr(acac)_4_ in bulk at 160 °C/15 min (No. 2, Table 2).

In a previous study, six different metal acac-complexes were used as catalysts in ROPs conducted in bulk with l-lactide as the monomer at temperatures up to 160 °C.^43^ Bu_2_Sn(acac)_2_ and Zr(acac)_4_ were the only catalysts reactive enough to achieve complete conversion in less than 3 hours. Since GL is more reactive than l-lactide,^12,44–46^ the acac complexes of manganese(ii), nickel(ii), copper(ii), and vanadium(v) and Bu_2_Sn(iv) were studied again (Table 1). The Cu(ii) complex yielded a low melt viscosity after 2 hours, while all the other metal acac complexes produced crystalline PGA within 2 hours. However, only the Bu_2_Sn and Mn complexes yielded completely soluble PGA, enabling GPC and MALDI TOF mass spectrometry, as shown in Fig. 1 and 2. In summary, the higher reactivity of GL allowed for polymerization with more acac complexes than l-lactide.

The GPC measurements (Fig. 1) yielded *M*_n_ values of around 2100–2 3 00 g mol^−1^, which is three times higher than those obtained by zwitterionic polymerization catalyzed by pyridine. The weight-average molecular weights (*M*_w_) were so low that the dispersities (Đ) were only around 1.3. This result is remarkable and differs largely from the dispersities of around 10 found for the cyclic PGAs obtained *via* zwitterionic polymerization.^41^ These molecular weights are interesting for three reasons. First, they allow for detailed studies of crystallization kinetics. Second, since the PGAs were brittle, they allow for easy grinding and the resulting crystal powders may be useful for 3D-printing with laser sintering.^47^ Third, the low molecular weights mean that that the MALDI mass spectra are representative of most of the sample components, despite the low signal-to-noise ratio. The GPC elution curves of the Sn- and M-catalyzed PGA samples were identical and displayed a weak tendency toward bimodality (Fig. 1A). A slightly different shape, with again a trend towards bimodality, was also observed for PGAs prepared by means of Zr(acac)_4_ (Fig. 1B). The origin is most likely the same for all the polymerizations in this study. Since PGA is one of the most rapidly crystallizing polymers, crystallization starts before conversion is complete, whereupon the catalyst concentration rises in the remaining melt and modifies the polymerization kinetics.

The MALDI mass spectra of the Sn- and the Mn-catalyzed PGAs (Fig. 2) were nearly identical and displayed exclusively peaks of cycles with equal intensity of even- and odd-numbered species (Fig. 2A). As is typical for PGA, the signal-to-noise ratio was poor, so that the mass peaks were only detectable up to *m*/*z* 3500. This low S/N ratio results from the densely packed PGA chains combined with strong electronic interchain interactions. When the less densely packed l-lactide was polymerized with Bu_2_Sn(acac)_2_ under identical conditions, the peaks of cycles were detectable up to *m*/*z* 9000 in the spectrum of the virgin sample and up to *m*/*z* 18 000 after fractionation, indicating that cycle formation was not limited to oligomers.^48^

Differential scanning calorimetry (DSC) measurements revealed melting endotherms and melting enthalpies within the range previously observed for cyclic PGAs with other cyclic catalysts or *via* zwitterionic polymerization.

### Polymerizations with Zr(acac)_4_

For the polymerizations with Zr(acac)_4_, the temperature, time and monomer/catalyst ratio were varied (see Table 2). The Zr(acac)_4_-catalyzed REPs were faster than those catalyzed by Bu_2_Sn(acac)_2_ or Mn(acac)_2_ and at 160 °C, 5 min sufficed to achieve nearly complete conversion. Crystallization became detectable after 10 min, even at a GL/Cat ratio of 2000/1.When the time was varied from 5 min to 2 h at a GA/Cat ratio of 500/1, an increase of *M*_n_ and *M*_w_ was detectable up to 30 min, but a slight decline occurred after 2 h. This trend is typical of a rapid, kinetically controlled polymerization followed by slower thermodynamically controlled equilibration reactions (*e.g.* ring–ring equilibration). However, this minor variation in molecular weight did not influence the thermal properties such as *T*_m_ and Δ*H*_m_. Compared to the Sn and Mn-catalyzed samples, a slight increase in all properties (*M*_n_, *M*_w_, *T*_m_ and Δ*H*_m_) was observed. Hence, Zr(acac)_4_ proved to be the most attractive acac catalyst with regard to reactivity and properties of the polymerization products. As expected for a REP mechanism and previously illustrated for l-lactide and cyclic tin catalysts, higher GL/Cat ratios did not yield any progress with regard to molecular weights and thermal properties.^49,50^ Polymerizations at lower temperatures were still successful at 130 °C but not at 100 °C (No. 6 and 7, Table 2).

**Table 2:** ROPs of glycolide catalyzed by Zr(acac)_4_^a^

Exp. no.	GL/Cat	Temp. [°C]	Time [min]	*M*_n_ [g mol^−1^]	*M*_w_	Dispersity *Đ*	*T*_m_ [°C]	Δ*H*_m_ [J g^−1^]
1A	500/1	160	5	2100	3600	1.7	218.8	106.4
1B	500/1	160	15	2300	4000	1,7	219.4	107.0
1C	5001	160	30	3600	6500	1.8	118 + 124	108.1
1D	500/1	160	120	2800	5800	2.0	218.3	110.7
2	250/1	160	15	2100	3400	1.6	218.2	115.1
3	1000/1	160	15	2600	5700	2.1	218.3	104.7
4	2000/1	160	15	2300	4000	1.7	218.2	107.3
5	500/1	130	30	2500	17 300	7.0	223.6	107.6
6	500/1	100	30	low conversion	—	—	—	—
7	100/1	100	30	Incomplete conversion	—	—	—	—

a Calculated with Δ*H*^0^_m_ = 206 J g^−1^.

The MALDI mass spectra revealed the following trends: At 160 °C and for short times (5 and 15 min), odd- and even numbered cycles were formed in equal quantities, and their molecular weight distribution roughly followed the most probable distribution as demonstrated in Fig. 1B.

**Fig. 3:**
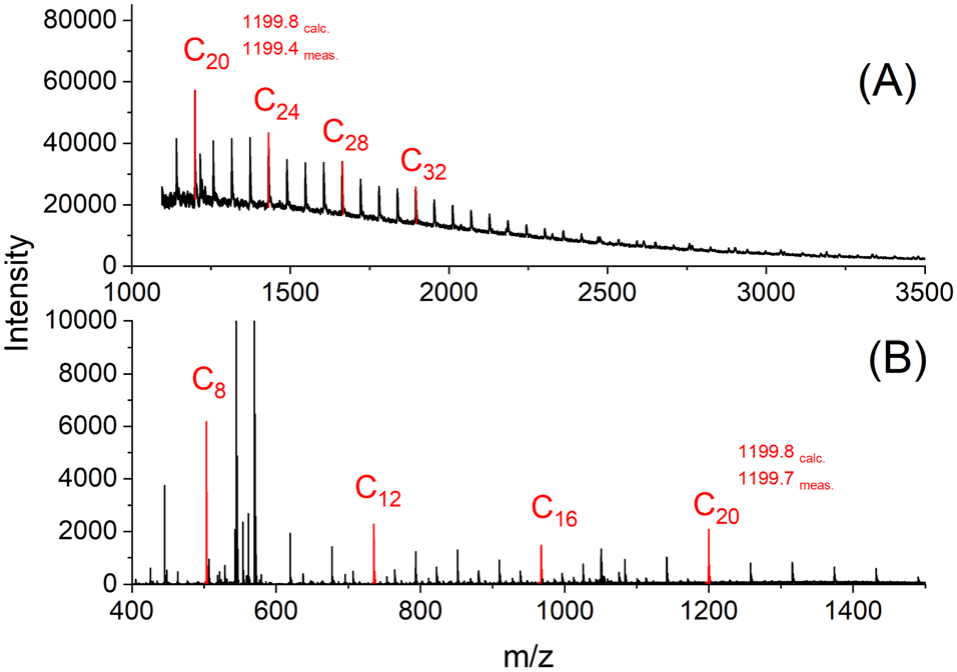
cPGA prepared with Zr(acac)_4_ (GL/cat = 500/1) at 160°C/0.5 h (No. 1C, Table 2): (A) MALDI TOF mass spectrum, (B) ESI TOF mass spectrum.

**Fig. 4:**
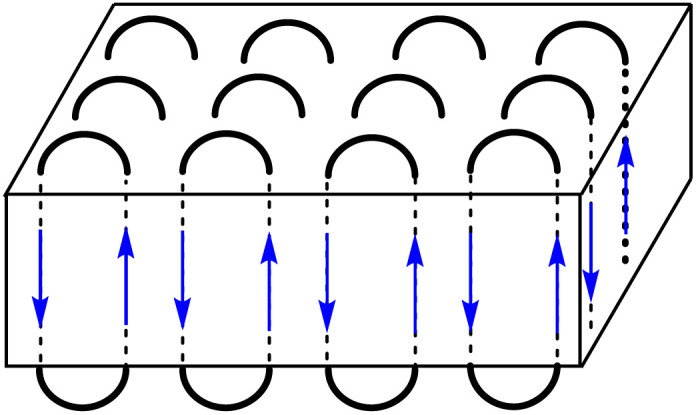
Schematic presentation of an extended-ring crystal.

**Fig. 5:**
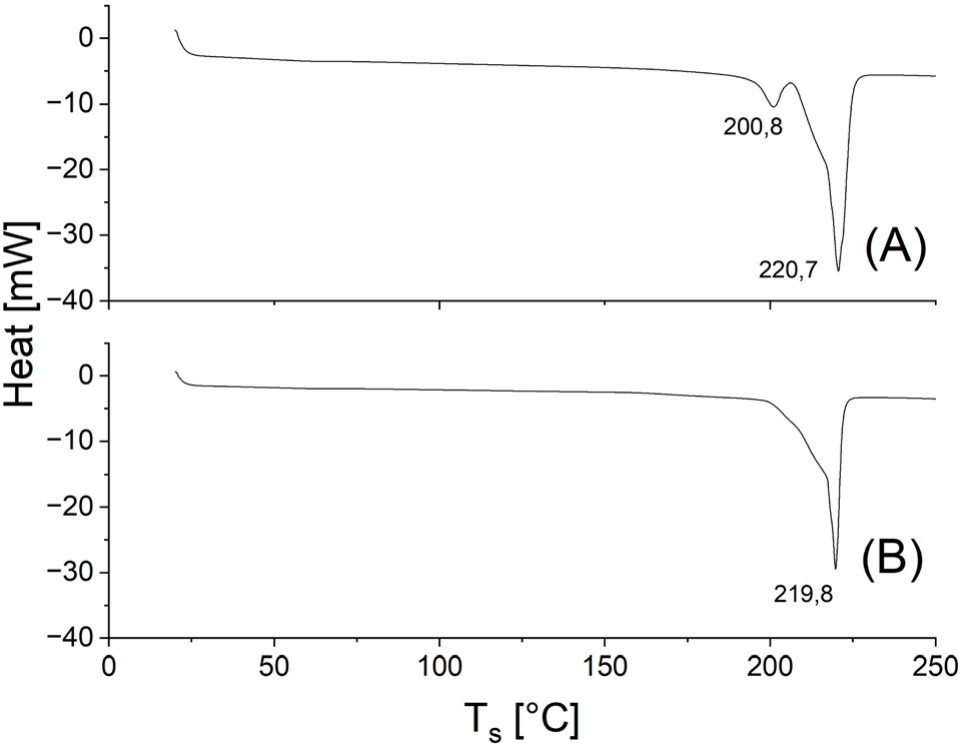
DSC heating traces of PGAs prepared with Zr(acac)_4_ at 160 °C: (A) after 5 min No.1A, Table 2): and (B) after 2 h (No.4, Table 2).

However, after 30 min a modification of this distribution emerged because the intensity of the peaks of certain even-numbered cycles increased (Fig. 3A). This trend continued down to the cyclic octamer as demonstrated by the ESI TOF mass spectrum in Fig. 3B. Starting with the cyclic octamer, every fourth cycle achieved a higher intensity up to a polymerization degree of 32. This type of mass distribution was observed to be even more pronounced for all cyclic PGAs prepared with tin catalysts at 160 °C.^39,40^ It was also observed, when a cPGA with a most probable distribution was annealed in the presence of SnOct_2_.^41^ Since the tin catalysts are more reactive than Zr(acac)_4_, they can easily catalyze transesterification reactions in the solid state. Under thermodynamic control, the most stable type of crystallites is formed, namely extended-ring crystallites (Fig. 4). The DSC measurements also indicate the existence of transesterification reactions, as two endotherms were detectable after short reaction times (see Fig. 5A). Upon annealing, the low-temperature endotherm disappears and forms a shoulder of the main endotherm (Fig. 5B).

The extended-ring crystallites represent a thermodynamic optimum, because they lack defects inside their crystal lattice and possess a rather smooth surface covered by loops of similar size. As shown previously, the length of the extended-rings agrees with the thickness of the crystallites calculated from SAXS measurements.^39^ All of these phenomena were previously observed in cyclic poly(l-lactide)s.^51,52^

**Fig. 6:**
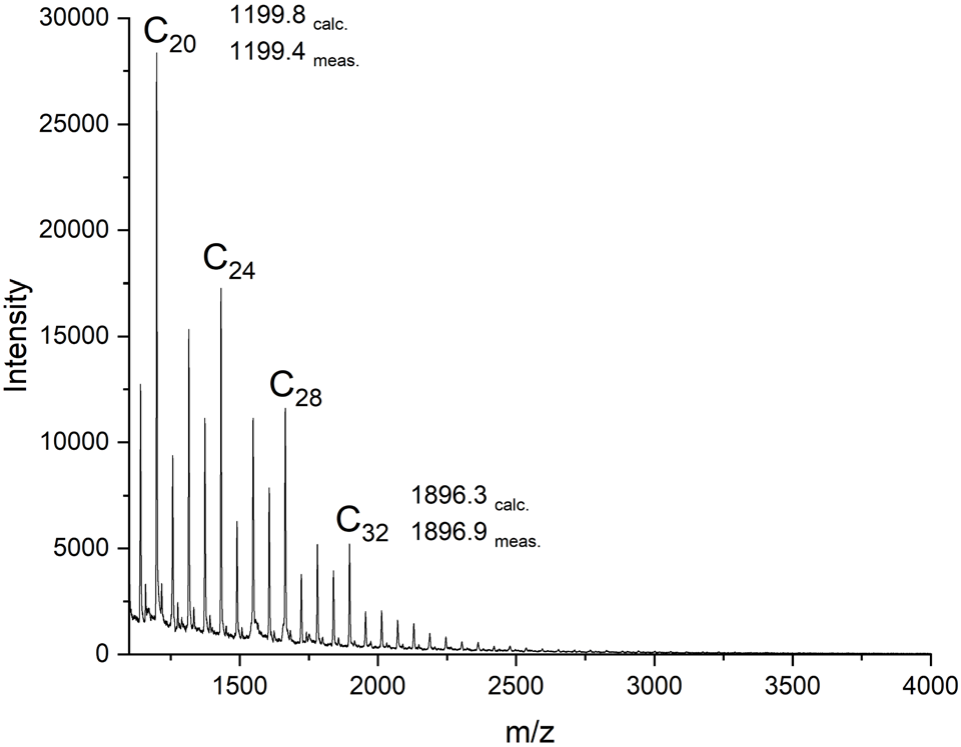
MALDI TOF mass spectrum of cPGA prepared with Zr(acac)_4_ in bulk at 130 °C/0.5 h (No. 5, Table 2).

Interestingly, another mass distribution was detected in the MALDI mass spectra of the cPGA prepared at 130 °C for 30 min. As demonstrated in Fig. 6, this sample displayed a strictly alternating sequence with regard to the intensity of even and odd cycles. This type of mass peak distribution is also been observed in poly(-lactide) prepared through alcohol-initiated polymerizations of LA at 130 °C or below. This sequence of mass peaks indicates that the initially formed even-numbered, chains or cycles were not completely equilibrated by transesterification. In summary, REPs catalyzed by Zr(acac)_4_ may yield three types of mass distributions, depending on temperature and time. With more reactive tin catalysts, only mass distributions showing the “saw-tooth pattern” have been observed thus far.

### X-ray measurements

**Fig. 7:**
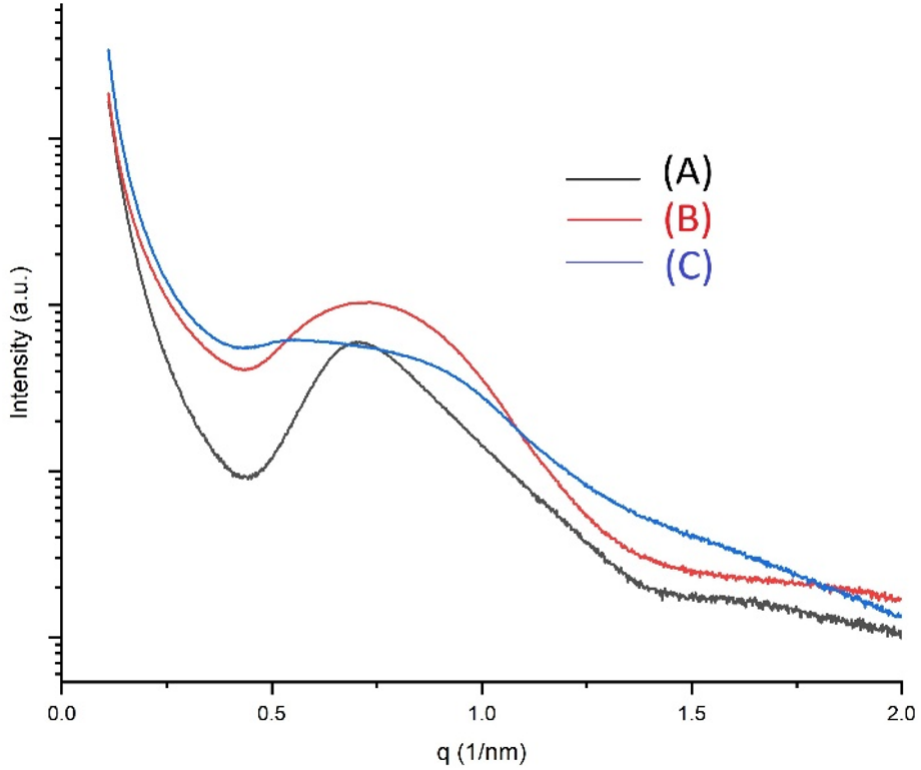
SAXS curves of cyclic PGAs: (A) No. 4, Table 1 or 3, (B) PGA prepared with pyridine at 120 °C ad GL/Py 100/1, (C) prepared with pyridine at 120 °C and GL/Py = 200/1.

The SAXS measurements yielded only one broad reflection, which corresponds to an *L*-value in the range of 7–8 nm (exemplarily illustrated in Fig. 7A). All samples are listed in Table 2. The SAXS curves all show broad reflections indicate a low degree of the three-dimensional order in the lamellar crystallites and amorphous phase. This low order is even more pronounced for PGAs prepared by zwitterionic polymerization in pyridine at 120 °C as demonstrated by the SAXS curves (B) and (C). *L*-values in the range of 7–8 nm are consistent with those found for previously synthesized high- and low-molecular weight cPGAs based on sharper reflections.^39–41^ It should be noted that all previous measurements, including those of PGAs prepared *via* polycondensation, have demonstrated that the *L*-values of PGAs are rather insensitive to variations in reaction conditions, in contrast to polylactides. Therefore, the absence of sharp reflections does not result in a significant loss of information regarding the crystal thickness.

**Fig. 8:**
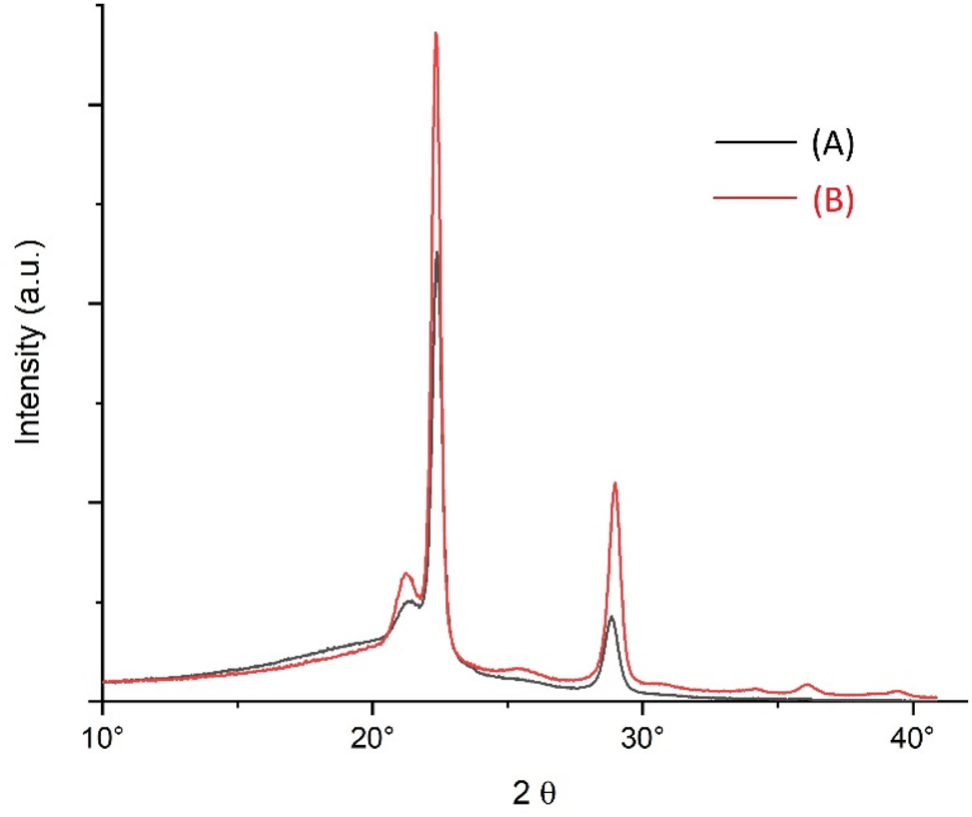
WAXS powder patterns of cyclic PGAs: (A) No. 1C, Table 1 (and Table 3), (B) No. 2, Tables 1 and 3

The WAXS powder patterns, two of which are presented in Fig. 8, displayed all reflections known from previous studies of other researchers.^51,53^ Furthermore, the WAXS patterns of all samples in this study were identical with regard to the position and relative intensity of the reflections. Therefore, it can be concluded that the cyclic PGAs prepared in this study crystallized in the same crystal lattice as the previously studied linear PGAs. Since these measurements included two samples of cPGAs prepared *via* zwitterionic polymerization, the above conclusion also applies to cPGAs with the lowest reported molecular weights.

The WAXS powder patterns revealed slight differences in crystallinity. These measurements were of interest for comparison with the crystallinities calculated from the DSC measurements. Calculating crystallinities requires the melting enthalpy of an ideal crystal (Δ*H*^0^_m_) for comparison. Unfortunately, three different values of Δ*H*^0^_m_ were reported in the literature: 206,^14,54^ 191 (ref. 10) and 183 J g^−1^.^55^ In previous publications the authors used the highest value, because it was determined by two research groups.^14,53^Table 3 shows the crystallinities of most PGA samples prepared with Zr(acac)_4_ calculated using all three literature values of Δ*H*^0^_m_ and compared with the crystallinities derived from the WAXS patterns.

**Table 3:** Comparison of crystallinities of PGAs prepared by means of Zr(acac)_4_

Exp. no.	GL/Cat	*T*_m_ [°C]	Δ*H*_m_ [J g^−1^]	Crystallinity [%]			
				a	b	c	d
1A	500/1	218.8	106.4	51	55	57	50
1C	500/1	118 + 124	108.1	53	56	59	42
1D	500/1	218.3	110.7	54	58	61	52
2	250/1	218.2	115.1	56	60	63	55
3	1000/1	218.3	104.7	50	55	57	47
4	2000/1	218.2	107.3	52	55	58	47
5	500/1	223.6	107.6	52	55	58	42

a calculated with Δ*H*^0^_m_ = 206 J g^−1^.

b calculated with Δ*H*^0^_m_ = 191 J g^−1^.

c calculated with Δ*H*^0^_m_ = 183 J g^−1^.

d calculated from WAXS powder patterns.

To properly interpret these results, consider that the WAXS method is not highly accurate, with a margin of error of ±5% relative to the measured value. Furthermore, these low-molecular weight PGAs may contain a significant fraction of small, imperfect crystallites that do not fully contribute to X-ray scattering but do contribute fully to DSC measurements. Nevertheless, the WAXS data clearly show the best agreement with the crystallinities calculated with the highest value. Thus, the authors' use of Δ*H*^0^_m_ of 206 J g^−1^ for evaluating all their DSC measurements is justified.

## Conclusions

The results obtained in this study suggest that metal acetylacetonate complexes, particularly Zr(acac)_4_, are effective catalysts for preparing cyclic PGAs in bulk. With Zr(acac)_4_, nearly 10% conversion can be achieved in 15 minutes at 150 °C. Only low molecular weights can be achieved, but in combination with a rather low dispersity. PGAs with this profile have not been synthesized by other catalysts. While these cyclic PGAs do not have useful mechanical properties, they may be of interest for academic studies, such as crystallization kinetics, solid state chemistry (*e.g.* formation of monodisperse extended-ring crystals), and exploring their potential use in 3D printing processes, such as selective laser sintering (SLS).

## Author contributions

SMW – investigation, data curation, methodology, validation, visualization, writing – review & editing; AM – investigation, methodology, visualization, writing review & editing; JM – investigation, methodology, visualization, writing review & editing, HRK – conceptualization, investigation, methodology, supervision, writing – original draft.

## Conflicts of interest

There are no conflicts to declare.

## Data Availability

All data will be made available on Zenodo at DOI https://doi.org/10.5281/zenodo.17826682.
